# Epigenetics in Food Allergy and Immunomodulation

**DOI:** 10.3390/nu13124345

**Published:** 2021-12-01

**Authors:** José A. Cañas, Rafael Núñez, Anyith Cruz-Amaya, Francisca Gómez, María J. Torres, Francisca Palomares, Cristobalina Mayorga

**Affiliations:** 1 Allergy Research Group, Instituto de Investigación Biomédica de Málaga-IBIMA, 29009 Málaga, Spain; joseantonio.canas@ibima.eu (J.A.C.); rafnunser@gmail.com (R.N.); anyithcruz@gmail.com (A.C.-A.); francis.palomares@gmail.com (F.P.); 2 Andalusian Centre for Nanomedicine and Biotechnology-BIONAND, 29590 Málaga, Spain; mjtorresj@uma.es; 3 Allergy Clinical Unit, Hospital Regional Universitario de Málaga, 29009 Málaga, Spain; paquigomez.p@hotmail.com; 4 Medicine Department, Universidad de Málaga-UMA, 29010 Málaga, Spain

**Keywords:** epigenetic mechanisms, food allergy, environmental factors, DNA modification

## Abstract

Food allergy (FA) is an increasing problem worldwide and, over recent years, its prevalence is rising in developed countries. Nowadays, the immunological and cellular processes that occur in the allergic reactions are not fully understood, which hampers the development of in vitro diagnostic tools and further treatment options. Moreover, allergic diseases could be reinforced by environmental exposure and genetic modifications. Gene expression can be controlled by different epigenetic mechanisms like DNA methylation, histone modifications, and microRNAs. In addition, several environmental factors such as dietary components (vitamin D, butyrate, folic acid) are able to regulate this epigenetic mechanism. All these factors produce modifications in immune genes that could alter the development and function of immune cells, and therefore the etiology of the disease. Furthermore, these epigenetic mechanisms have also an influence on immunomodulation, which could explain sustained responsiveness or unresponsiveness during immunotherapy due to epigenetic modifications in key genes that induce tolerance in several FA. Thus, in this review we focus on the different epigenetic mechanisms that occur in FA and on the influence of several dietary components in these gene modifications.

## 1. Introduction

Food allergy (FA) is a great problem for public health systems. Its prevalence is increasing in developed countries, reaching rates of around 8% in children and 3% in adults [1]. FA also affects patients’ quality of life, in part due by anxieties associated with fear of adverse reactions associated with accidental ingestion of allergenic food. FA displays a broad spectrum of clinical manifestations and their immune responses can be immunoglobulin E (IgE)-dependent or non-IgE-dependent. There is no precise in vitro diagnosis to confirm FA. Besides, although oral immunotherapy (OIT) is currently widely accepted, there is still a lack of additional, more precise treatment options. There are some immunomodulatory treatments, like allergen specific immunotherapy (AIT), that modify the development of the disease towards food tolerance. Nonetheless it is necessary to improve allergy testing practices in order to reach a more precise diagnosis and to consequently improve treatments. In this sense, identifying clinical allergy biomarkers may be useful in case of lack of evident clinical history.

Development of FA can be related to some concepts such as the influence of genetic, epigenetic, environmental, and nutritional factors [2], which seem to be involved in its etiology. Genetic studies have shown that there are hundreds of genes in FA that are associated with food-specific IgE, providing strong evidence that genome can determine the complex trait of IgE-mediated FA [3]. This phenomenon would be reinforced by the effect of environmental exposure in the genome, which would alter the expression of genes associated with the immunopathology of the disease and, therefore, its etiology [4]. Furthermore, epigenetic alterations may explain why individuals with different genotypes are diversely affected by environmental variations.

**Figure 1:**
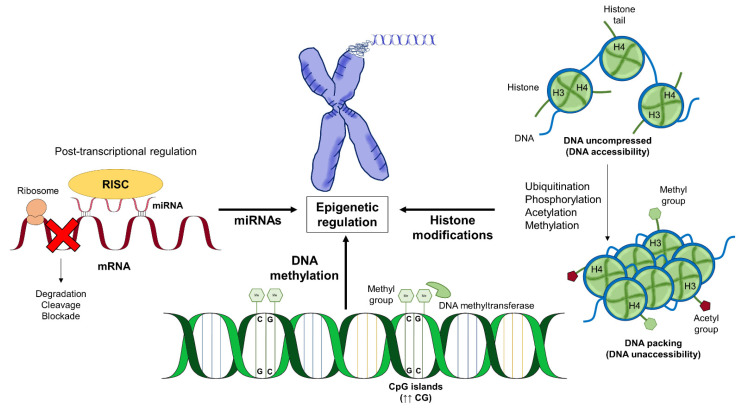
Gene expression regulation can be exerted by three different epigenetic mechanisms: histone modifications, DNA methylation, and miRNAs. DNA methylation involves the addition of a methyl group to a cytosine in CpG dinucleotide sequences (CG), and CG rich regions are known as CpG islands. Histones can be modified by ubiquitination, phosphorylation acetylation, and methylation, producing DNA packing. miRNAs are non-coding RNAs that bind to mRNA and block protein translation. RISC, RNA-Induced Silencing Complex.

The models of gene–environment interactions are associated with the epigenetic mechanisms of gene regulation [5], which can be exerted by three different mechanisms (Figure 1): (i) DNA methylation involves the addition of a methyl group to a cytosine to form a 5-methylcytosine by DNA methyltransferases (DNMTs). In particular, mammalian genomes are mainly methylated in CpG dinucleotide sequences (CG), and CG rich regions are known as CpG islands [6,7]. Such landmarks are highly unmethylated and are present in the vast majority of gene promoters. The increase in methylation of these CpG islands predominantly reduces gene expression of the adjacent gene [8,9]. In this manner, allergic pathological processes can alter gene expression by switching methylation patterns of CpG islands, and vice versa. (ii) Histones can be modified in multiple ways, as ubiquitination, phosphorylation, acetylation, and methylation, with the two latter modifications being the most extensively studied and associated with changes in gene expression [10]. Histone modification controls DNA accessibility, compressing, or unpacking chromatin, and therefore establishes another level of gene expression regulation [11]. Increased acetylation levels in *N*-terminal lysine residues of histones H3 and H4 positively regulate chromatin decompression; hence, deacetylation favors chromatin compression, decreasing gene expression [12,13,14]. However, histone methylation can lead to activation or inactivation depending on target residue, regularly lysines or arginines in H3 or H4 histones [15,16]. (iii) Non-coding RNAs are non-translatable transcripts that can potentially regulate gene expression. Among them, the most noteworthy are microRNAs (miRNAs) [17,18,19]. These small non-coding RNAs are 19–24 nt in length and are involved in post-transcriptional gene regulation through the interaction between miRNA and a target mRNA [20,21,22,23]. On top of this, translation disruption of specific mRNAs, miRNAs, could affect histone modifications and DNA methylation [24,25,26,27].

In different type of FAs, several studies have described different epigenetic mechanisms involved in the immunological response of the disease. In fact, peripheral blood mononuclear cells (PBMCs) and purified T cell populations from FA individuals have diverse DNA methylation patterns compared to non-allergic subjects, which affects genes involved in T cell activation, mammalian target of rapamycin signaling pathway, and the mitogen-activated protein kinase (MAPK) pathway, with these effects being more marked in persistent FA [28]. In fact, several studies developed by Martino et al. observed that immunological pathways modified by gene-environment interactions induce epigenetic dysregulation in the early stages of T cell activation in childhood FA [29,30]. They studied DNA methylation to delineate epigenetic modifications in activated anti-CD3/anti-CD28 bead-bound T cells in egg-allergic patients and non-atopic controls, and found a distinct DNA methylation profile for genes involved in metabolic regulation in egg allergy. The same group observed that 96 CpG sites predicted clinical reactivity to food challenge in children. These sites coincided with 73 protein-coding genes that mostly belong to the MAPK canonical pathway. Besides, other studies suggested that epigenetic modulation may play a key role in allergic sensitization and inflammation [31,32], for instance in the use of DNA methylation profiling in specific CpG sites that predict allergic sensitization. Additionally, other studies of FA in animal models shows that epigenetic regulation of mast cell activation during immune responses may happen through altered histone acetylation, and the exposure to food substances could trigger epigenetic modifications, modulating the function of mast cells [33]. In addition to these studies, in the following section we will describe in depth those related to cow’s milk and peanut allergy.

Moreover, we know very little about epigenetics and immunomodulation. Several studies determine that epigenetic control can mediate the regulatory response in FA [34,35]. In this sense, the epigenetic modifications may be the crucial to explain how environmental factors modulate and modify gene expression towards a regulatory pattern. Related to this, specific treatments as specific allergen immunotherapy, nutrients like vitamin D, as well as food fibers and folic acid lead to epigenetic changes, which are associated with immune tolerance. This provides new approaches to identify biomarkers of response that can help in the diagnosis and treatment of FA. However, the epigenetic changes of FA differ between studies and more research is needed, including exploratory research to identify the different epigenetic mechanisms involved in FA. Despite this, the studies that we describe in this review represent new lines of research that open up new horizons for the management of FA.

## 2. Epigenetics in Food Allergy

Recent studies have revealed that epigenetics and environmental exposures contribute to the development of FA [36], and reveal new mechanistic insights into disease. In this section, we will describe the most recent evidences on the epigenetic modifications in the most frequent FA, cow’s milk, and peanut allergy.

### 2.1. Cow‘s Milk Allergy

Recent studies have elucidated the epigenetic changes in cow’s milk allergy (CMA), which is one of the most common FA in children. A study by Canani et al. showed that DNA methylation of IFN-γ, IL-4, and IL-5 cytokine genes and *FOXP3* affects CMA disease, suggesting that the miRNome could be implicated in the pathogenesis of CMA [37]. The results showed that miR-193a-5p regulated IL-4 gene expression and may play a role in IgE-mediated CMA, suggesting that this miRNA could be a novel diagnostic and therapeutic target for this common form of FA in children [38]. In this regard, miR-193a-5p resulted to be the most downregulated miRNA in allergic children to cow’s milk compared to healthy controls. Its role in IgE-mediated allergic response is also supported by functional data on IL-4 RNA expression and protein synthesis. The authors also confirmed that children who developed a tolerant response reached a miR-193a-5p level similar to controls, suggesting that the expression of this miRNA could be influenced by the disease state and could become a biomarker for monitoring the disease course.

Previously, the same authors described that the tolerance acquisition in children with IgE-mediated CMA was characterized by a distinct Th1 and Th2 cytokine gene DNA methylation pattern and epigenetic regulation of the *FOXP3* gene [37,39]. In addition, this study showed different *FOXP3* demethylation patterns compared to CMA children with active disease to those with recent immune tolerance acquisition [39]. In fact, these studies have been reinforced by others that confirmed that dietary factors such as dietary fibers play a fundamental role in the regulation of epigenetic mechanisms [40]. In this sense, a significant difference in DNA methylation of Th1/Th2 cytokine genes was observed in tolerance-induced children after treatment with hydrolyzed casein formula with the probiotic *Lactobacillus rhamnosus GG*, with respect to subjects who received other formulas, like soy formula [41]. Regarding diet, a study suggested that the tolerogenic mechanisms elicited by different formulas commonly used for CMA management could be induced through an epigenetic modulation of *FOXP3* gene [42]. In other pilot study in CMA, the hypermethylation was found in regions of *DHX58*, *ZNF281*, *EIF42A*, and *HTRA2* genes from the CMA group compared to control children (healthy controls), whereas this was not observed in the tolerant group (children had proven CMA, but who were already tolerant to cow’s milk protein) [43]. 

On the other hand, in an animal model study, the authors assessed if raw cow’s milk could induce tolerance to unrelated, non-milk, food allergen. The raw milk decreased allergic symptoms compared to processed milk. The epigenetic analyzed, histone acetylation of T-cell genes, determined that raw milk was able to modulate gene expression through epigenetic mechanisms targeting histone marks on T cell-related genes. The activation of T cell-related genes could induce such tolerance, suggesting that epigenetic modifications contribute to the allergy-protective effect of raw milk [44]. The same authors determined, in another study, a decrease in histone acetylation levels at Th1 cells and regulatory loci after induction of FA [45].

### 2.2. Peanut Allergy

Allergic reactions to peanut are the most common cause of fatal food-related anaphylaxis. Currently, from a genetic point of view the research is focused on the identification of genomic biomarkers of clinical utility for the diagnosis of peanut allergy. In this sense, Hong et al. performed a well-defined genetic study in FA that analyzed the genome-wide including different subtypes (peanut, milk, and egg) of FA patients in the US [46]. This genetic study identified peanut allergy-specific loci in the HLA-DR and -DQ gene regions in patients of European ancestry. They replicated these associations in an independent sample of European ancestry, with the results being supported by meta-analyses in the discovery and replication samples. The peanut allergy specific loci in the *HLA-DR* and *-DQ* gene regions are single-nucleotide polymorphisms (SNPs), with these SNPs being associated with differential DNA methylation levels at multiple CpG sites. The study suggested that the *HLA-DR* and *-DQ* gene region likely poses a significant genetic risk for peanut allergy [46]. Another study revealed that DNA methylation profiling on PBMCs from peanut allergic and non-allergic patients in 96 CpG sites, predicting clinical outcomes [47]. They determined several potential new genes associated with peanut allergy, such as *BDNF* (neurotrophin) and *SERPINE1* (serine protease inhibitor), showing a new research line to elucidate the molecular basis of peanut allergy. Furthermore, a high similarity between peanut allergic- and non-allergic patients was observed in monozygotic twins compared to randomly-paired genetically unrelated individuals. This indicates that 12 DNA methylation signature-associated peanut allergy was genetically influenced [47]. Another study described different DNA methylation profiling in Th1 and Th2 cells comparing non-allergic and peanut allergic patients. This study showed a decrease in DNA methylation levels in genomic regions associated with *IL4* and *IL2*, and an increase in DNA methylation levels in the genomic region of *IL12B* [48]. Consistent with this study, others found that DNA methylation levels decreased significantly for the gene *IL1B* and *IL6* in peanut-allergic patients compared to non-allergic, which suggests a relation between innate immune system epigenetic regulation and FA [47], expanding mechanistic understanding the severity of reactions in peanut allergy. Another study carried out an integrated transcriptomic and epigenetic analysis of peanut-allergic children. The authors integrated whole blood transcriptome and CD4^+^ T-cell epigenome profiles to identify molecular signatures of reaction severity. The results identified *NFKBIA* and *ARG1* genes as potential targets of the severity of the reaction in peanut and other allergies [49].

Recent data show the role of B-cell dysfunction in FA. A study evaluated epigenetic changes in these cells, analyzing epigenetic and transcriptomic patterns in purified B cells from patients with FA, comparing single-food-allergic (peanut only), multi-food-allergic (peanut and other food), and non-allergic (control) patients. The results suggested that B cells from peanut patients showed specific epigenetic and gene expression differences relative to non-allergic patients. Furthermore, these results suggested that single-peanut-allergic and multi-food patients possessed unique group-specific epigenetic signatures involving differential regulation of B- and T-cell development, B-cell lineage determination and tumor growth factor beta (TGF-β) signaling pathway, highlighting the phenotypic and molecular distinction of the multi-food clinical group from single-peanut-allergic patients [50]. However, another work has shown promising data regarding gene expression differences between diverse FA endotypes and control patients. They observed a correlation between *TLR2* methylation and *TLR2* and *CD14* expression in nut-allergic children, indicating that the promoter region methylation is crucial for the overall regulation of expression of these genes [51].

In a murine model, the authors studied whether maternal atopy was a risk factor for the development of peanut allergy. The study determined whether offspring from peanut allergic mothers were more susceptible to peanut allergy than offspring from naive mothers in a murine model; and if so, whether the susceptibility was related to Th2-biased epigenetic alterations. Maternal peanut allergy induced epigenetic alteration of the *IL4* promoter in their offspring, which correlated with a Th2 biased responses (IL-4 and IgE production) [52]. Another animal study defined the mechanisms of tolerance induction to peanut protein and peanut allergy prevention. Here, different doses of peanut extract (PE) were administered to mouse pups every day for 2 weeks prior sensitization and challenge to peanut [53]. The results showed that the feeding with high (but not low) doses of peanut prior to sensitization induced tolerance, with an increase in the CD4^+^CD25^+^FoxP3^+^ cell percentages in mesenteric lymph node (MLN), in the FoxP3 mRNA and protein expression in CD4^+^ cells from MLN or jejunum. In addition, the feeding with high doses of peanut prior to sensitization decreased CD3^+^CD4^+^IL13^+^ cells and CD3^+^CD4^+^IL17^+^ cells percentages in MLN; and reduced *IL13* and *IL17A*, while increasing *TGFB* mRNA expression in the jejunum. The percentage of CD103^+^ DCs in MLN increased significantly. Treg suppression was observed to be antigen specific and the *FOXP3* methylation increased in PE sensitized mice, while these levels were significantly lower in tolerized mice [53].

## 3. Epigenetics in Food Allergy Immunomodulation

In the last few years several studies have addressed the role of environment and dietary factors in FA. Specifically, epigenetic modifications may regulate the susceptibility of FA throughout expression of key genes. This fact could imply loss of immune tolerance and initiation of FA. On the contrast, epigenetic mechanisms can also restore the dysregulated immune imbalance of FA and behave as immunomodulatory factors in this allergic disease. In this part of the review, we focus on epigenetic immunoregulation towards tolerance and how several dietary components can produce these changes.

### 3.1. Epigenetics and Immunomodulation

In the last decade, many works have demonstrated the role of epigenetics in the regulation of allergy development and during immunotherapy responses. In 2014, Syed and collaborators demonstrated that DNA methylation could play a role in sustained responsiveness in peanut-allergic patients with OIT through hypomethylation of *FOXP3* gene and increase in antigen-induced Treg cell functions [54]. They demonstrated that OIT produced *FOXP3* hypomethylation, increasing tolerance to peanut, and the sustained unresponsiveness to OIT matched with higher methylation of *FOXP3*. Moreover, they followed 20 OIT participants successfully desensitized and found that *FOXP3* methylation was lower in those participants than in peanut-resensitized subjects, suggesting that an increase in methylation on CpG in the *FOXP3* locus could play an important role in resensitization [54]. Later, in 2018, Mondoulet et al. observed that epicutaneous immunotherapy (EPIT) modulated changes in DNA methylation of two key genes for immunomodulation (hypermethylation of *GATA3* and hypomethylation of *FOXP3*) in peanut-sensitized mice [55]. This was also previously demonstrated in a milk-allergic murine model, where EPIT enhanced methylation of the promoter region of *GATA3*, inducing protection against further Treg-cell-dependent sensitization [56]. This study demonstrated that this fact could be used as potential biomarker to identify patients that have responded to EPIT, which had also been suggested by Martino et al. [32]. Moreover, another study performed in allergic patients who received sublingual immunotherapy (SLIT) against timothy grass and dust mite suggested that SLIT promoted the increase in memory Treg cells by reducing DNA methylation of CpG sites within the *FOXP3* locus [57]. All these studies have demonstrated the importance of induced tolerance by immunotherapy through DNA methylation of Treg cells [53].

Currently, there are no available epigenetic biomarkers. Nonetheless, some previous studies have suggested that DNA signature methylation can be used to distinguish allergic patients from non-allergic subjects [47]. Therefore, all these results could indicate that DNA methylation could be used as marker of clinical relevance for the success of immunotherapy. However, more studies in this field are needed to elucidate it.

### 3.2. The Role of Vitamin D Epigenetics in Food Allergy

Vitamin D is an essential compound to maintain the homeostasis of several important functions of the organism, such as calcium and phosphorus absorption, and maintaining normal functions of immune system. Vitamin D active forms can be synthetized from two fat-soluble compounds: pre-vitamins D2 and D3. The first one is acquired from food and dietary supplements, and the second one is synthetized in the skin from the 7-dehydrocholesterol by UVB ray’s exposition (sunlight). Then, in the liver, vitamin D is metabolized to 25-hydroxyvitamin D (25(OH)D3), which will be converted to 1,25-dihydroxyvitamin D (1,25(OH)2D), which is the active form of vitamin D [58]. This last conversion occurs in several tissues and cell types including kidney and immune cells (T cells and DCs) [59].

Related to FA, investigations about the relationship between vitamin D and FA have increased during the past decade. In these studies, vitamin D deficiency has been linked to high risk of several allergic diseases, including atopic dermatitis and FA [60,61,62,63], like peanut and egg allergy [64]. However, high levels of vitamin D can also affect negatively since, as demonstrated in a recent randomized controlled trial, there was an increased risk of allergic sensitization and CMA in infants with higher vitamin D supplementation [65].

There is much evidence linking vitamin D to the development of FA at the genetic level. Active vitamin D binds to vitamin D receptor (VDR) inside T cells and antigen-presenting cells, and modulates several genes involved in the immune response [70]. This immunoregulatory effect is performed by modulation of expression of relevant genes in FA, including cytokine encoding genes such as IFN-γ, tumor necrosis factor alpha, and IL-10 [68,71], recruiting active vitamin D-VDR complex to promoter region of these genes. In addition, VDR activation by 1,25(OH)2D produces DC-derived gene reprograming towards tolerogenic phenotype [72]. Another study demonstrated that low serum 25(OH)D3 levels (≤50 nmol/L) in infants were associated with high risk of developing FA (eleven more times in peanut allergy, four more times in egg allergy, and ten more times in multiple food allergies) [64]. On the other hand, Koplin and collaborators showed that a polymorphism (rs7041T allele) in vitamin D-binding protein increases the biological availability of serum vitamin D and, then, FA protection [73].

Moreover, vitamin D can act at cellular, molecular, genetic, and epigenetic level to regulate FA [51]. At cellular and molecular levels, it has been described that active form of vitamin D has direct and indirect effects on the function of immune cells, including T cells, DCs, and Tregs [66,67]. This is achieved through modulation of immune mediators, like pro- and anti-inflammatory cytokines, and IgE [68,69].

Returning to the aims of this review, it has been studied that vitamin D can also modulate FA by epigenetic regulation, as not all genes regulated by vitamin D have vitamin D response elements [74]. However, the role of vitamin D in epigenetic regulation is still unclear, and it has been rarely explored. In 2016, Junge et al. demonstrated the relationship between thymic stromal lymphopoietin (TSLP) and vitamin D levels from high cord blood at birth and in early infancy [74]. They observed that higher levels of 25(OH)D3 correlated with lower levels of methylation in TSLP enhancer region, resulting in a higher TSLP mRNA expression. Indirectly, the methylation and repression of several genes related to vitamin D function and metabolism, including *VDR*, *CYP2R1*, and *CYP24A1*, can induce FA [75]. These genes are susceptible to DNA methylation because they contain large cytosine-guanine islands. Zhu and collaborators showed that vitamin D deficiency in adolescents was associated with methylation changes in *DHCR7*, *CYP2R1*, and *CYP24A1* from leukocytes, which are crucial for vitamin D metabolism [76]. More evidences that vitamin D affects DNA methylation were shown by Anderson et al., demonstrating that maternal vitamin D supplementation during pregnancy and lactation alters DNA methylation in mothers and breastfed infants [77]. However, they did not show the short- and long-term biologic effects. Moreover, it has been studied that vitamin D deficiency in gestational status alters Th1/Th2 balance, decreasing IFN-γ production by methylation on the gene that encodes it [78]. The important role of vitamin D in DNA methylation has been observed in Treg cells. It is well known that stable levels of Tregs depend on *FOXP3* methylation; consequently, low levels of vitamin D reduce Treg population through change in methylation on *FOXP3*, increasing therefore the risk of FA [79]. In contrast, a negative correlation on Treg cells and vitamin D, showing that high levels of vitamin D could suppress Treg number through the methylation of *FOXP3* increasing the risk of FA development has been also reported [79]. Due to the existence of controversial studies, additional ones are required to deepen the role of vitamin D inducing immune tolerance and the risk of allergy development, specifically FA [80,81].

### 3.3. Butyrate

Butyrate has been involved in FA through epigenetic mechanism regulation. Butyrate is a short-chain fatty acid (SCFA) formed by microbial fermentation of dietary substrates like fiber, therefore it is produced in the colon as a carbon source for colonocytes. Butyrate is absorbed in the proximal and distal colon by both passive diffusion and active transport mechanisms. It is also important for the absorption of electrolytes by the large intestine, and plays a critical role in mucosal integrity and local and systemic metabolic function, stimulating regulatory immune responses. Notably butyrate may play a role in preventing certain types of diarrheas and it has shown beneficial effects on some colonic pathologies [82,83,84]. The attenuation of the deleterious immune response and hyperinflammation could be mediated by SCFA produced by the gut microbiota [85].

Interaction between intestinal contents and immune and non-immune cells bring on environment that promotes tolerance by the induction of IgA antibodies and Treg cells, which produce IL-10. Cell surface SCFA receptors are expressed on the gut epithelium and intestinal immune cells such as DC and Treg cells. One important aspect is the development of allergen-specific CD4^+^CD25^+^ T-reg-cells secreting IL-10 in the AIT of FA diseases. Moreover, butyrate influences intestinal CD103^+^ DC by stimulating the GPR109a cell surface receptor, which enables this tolerogenic DC subpopulation to induce proliferation and expansion of Treg cells in MLN [82,86,87,88]. This expansion of intestinal Treg cells is owing to butyrate promoting the acetylation of histones at the *FOXP3* gene inhibiting lysine deacetylase, protecting at the same time the FOXP3 protein from degradation through enhancing its acetylation, and this suppresses inflammation and untoward immune responses in intestinal tissues [89]. Additionally, in in vivo models, mice sensitized with allergen showed a higher anaphylactic symptom score and body temperature reduction compared to control mice. Then, they were exposed to an oral daily dose of butyrate and an inhibition of allergic response was observed. Moreover, it also reduced serum-IgE concentrations [90].

Besides, environmental factors produce DNA methylation and histone modification and induce FA [88]. Butyrate and other SCFAs, besides being histone deacetylase (HDAC) inhibitors, are also able to induce histone modifications in immune cells by behaving as acyl-CoA precursors. Therefore, SCFAs can be considered as molecules capable of regulating gene expression at the epigenetic level through modulation of the activity of both histone acetyltransferase and HDACs [91].

### 3.4. Methyl Group Donors Folic Acid

Exposure to certain nutrients, such as methyl donors, present in food can modify methylation patterns and lead to allergic diseases [92]. Specifically, methyl donors such as folate take part in one-carbon metabolism, a process that provides methyl groups for DNA or histones methylation among others [93]. Multiple observational studies reported associations between folic acid supplementation during pregnancy and children proneness to develop allergy [92,94], although properly controlled trials are still needed to demonstrate folic acid transgenerational proallergic effect. Moreover, several studies have shown a correlation between methylation changes in immune cells and FA in infants [29,32,37,43,95]. Consequently, folic acid supplementation during pregnancy may be a determinant factor in the development of allergic diseases in childhood and demands more specific studies.

## 4. Conclusions

In this review, we summarize the different available studies related to FA and epigenetic mechanisms. Apart from explaining these different mechanisms of gene regulation, we highlight how they can be modulated by other different factors such as vitamin D, butyrate, and folic acid. We described the interplay between environment and genetics, revealing how epigenetic modifications could mediate genetic susceptibility of FA. This explains why epigenetic modifications could be relevant in environmentally-mediated gene expression and lead to the loss of immune tolerance and, finally, to FA. However, the study of the role of epigenetics in FA is still scarce and needs to be completed. Until now, these studies have been limited to only a small number of individuals or to the evaluation the epigenetic mechanisms in the development of disease. Furthermore, the different studies of epigenetic modifications in FA describe the role of them in the differentiation of T cell lineages and their influence on the balance between distinct T cell populations. In addition, the epigenetic modifications are influenced by various environmental factors such as dietary components, which are food allergy-protecting. Therefore, the emerging epigenetic paradigm in FA is likely to provide new mechanistic insights into FA risk and development as well as shape the different therapeutic and preventive strategies.
